# Temporal Trend, Prevalence, Predictors, and Outcomes of Pericardial Diseases in Patients Undergoing Transcatheter Aortic Valve Repair

**DOI:** 10.7759/cureus.16083

**Published:** 2021-07-01

**Authors:** Kashyap Shah, Matthew Krinock, Harshith Thyagaturu, Rezwan Munshi, Ayushi Pandya, Sarah Falta, John Hippen, Michael Durkin

**Affiliations:** 1 Internal Medicine, St. Luke's University Health Network, Bethlehem, USA; 2 Internal Medicine, St. Luke’s University Health Network, Bethlehem, USA; 3 Internal Medicine, Bassett Healthcare Network, Bassett, USA; 4 Internal Medicine, Nassau University Medical Center, East Meadow, USA; 5 Public Health and Policy, Edward J. Bloustein School of Planning and Public Policy at Rutgers University, New Brunswick, USA; 6 Medicine, Lewis Katz School of Medicine, Philadelphia, USA; 7 Cardiology, St. Luke's University Health Network, Bethlehem, USA

**Keywords:** transcatheter aortic valve repair, pericardial disease, acute pericarditis, pericardial effusion, aortic stenosis

## Abstract

Background

Pericardial disease (PD) - acute pericarditis (AP) and pericardial effusion (PE) - is a rare complication of transcatheter aortic valve repair (TAVR) although its prevalence, predictors, and outcomes are not well studied.

Methods

We used the National Inpatient Sample (NIS) database to find patients who received TAVR between 2011 and 2018. TAVR patients were divided into two groups: with and without PD (AP and/or PE). The baseline characteristics between the two groups were compared using the Chi-square test and student t-test. Variables with a p-value of 0.20 or less from the univariate logistic regression were included in the multivariate logistic regression to find independent predictors of PD in TAVR patients.

Results

Out of 218,340 TAVR hospitalizations, 4323 (1.2%) had a concurrent diagnosis of PD. TAVR patients with PD were older (81 ± 7 vs 80 ± 6 years, p < 0.05), more likely to be females (62 vs 46%, p < 0.001), white (84.2 vs 82.9%, p = 0.83), and had a higher burden of comorbidities (Table 3). TAVR patients with PD had higher in-hospital mortality rate (12.3 vs 1.9%, p < 0.001), mean length of stay (8.4 vs 5.3 days, p < 0.001), and mean total hospital cost ($283,389 vs $224,544, p < 0.001). Age > 75, female sex, atrial fibrillation (Afib), atrial flutter (Aflutter), peripheral vascular disease (PVD), coagulopathy, cirrhosis, malnutrition, percutaneous coronary intervention (PCI), coronary artery bypass grafting (CABG), and pacemaker (PM) implantation were the independent predictors of PD in TAVR patients.

Conclusion

Older, white females with a higher burden of comorbidities and cardiovascular procedures are at higher risk of pericardial complications of TAVR procedure. Sex-based disparities in the prevalence of PD after TAVR is an area of further research. Careful selection of patients for TAVR is essential to reduce the burden of these complications.

## Introduction

Aortic stenosis (AS) is the most common valvular heart disease requiring surgical intervention and has a prevalence that continues to rise with aging of the general population [1]. Transcatheter aortic valve repair (TAVR) is a minimally invasive procedure for the treatment of severe AS. It is becoming an increasingly more utilized treatment option over traditional surgical aortic valve replacement (SAVR) in many patient populations [2]. The major driving factor to this trend is due to emerging data showing an overall decrease in mortality and procedure-related complications coupled with patients and physicians’ desire for the less invasive procedures [3]. TAVR was originally utilized for high surgical risk patients, and it showed superiority over SAVR in this population [4]. As further research continues, subsequent clinical trials showed TAVR non-inferiority to SAVR for both high and intermediate surgical risk patients [5]. During the most recent clinical trials, Placement of Aortic Transcatheter Valves (PARTNER) 3 and Evolut R, TAVR was shown to be a non-inferior and, in some cases, superior alternative to SAVR for low surgical risk patients due to decreased mortality and non-inferiority, respectively [2,6,7]. As the indications for TAVR continue to expand, several periprocedural and postprocedural complications have gained interest in recent years. Compared to SAVR, TAVR is associated with a lower risk of bleeding and atrial fibrillation (Afib) although the short-term risk of reintervention, paravalvular leak, valve thrombosis, and pacemaker (PM) implantation remains high [8]. Pericardial diseases (PD) - acute pericarditis (AP) and pericardial effusion (PE) - is an additional complication of TAVR as a part of post-cardiac injury syndrome (PCIS) although there is a paucity of data, which are limited to few case studies and review articles [9-11]. To elucidate this rare but important complication of TAVR, we analyzed an eight-year (2011-2018) trend of prevalence, predictors, and outcomes of PD in patients undergoing TAVR using the National Inpatient Sample (NIS) database. To our knowledge, this is a rare study to analyze this trend in TAVR patients using the NIS database.

## Materials and methods

Data source

We queried the NIS 2011-2018 database for our study. The NIS is a large, publicly available, all-payer national database with over seven million hospital stays in the United States each year [12]. The database contains both patient (age, sex, race, comorbidities, primary expected payer, etc.) and hospital-level (hospital location, bed size, teaching status, etc.) data. The comorbidities in the NIS database are registered as either International Classification of Diseases Clinical Modification, 9th Revision (ICD-9) or International Classification of Diseases Clinical Modification, 10th Revision (ICD-10) codes. Charlson comorbidity index is a method of categorizing comorbidities based on ICD codes [13]. Each comorbidity is assigned a weight (from 1 to 6) based on its adjusted effect on mortality and resource utilization [13]. All the weights are then added to calculate a Charlson score for each patient. Each hospital in the NIS database is considered either a rural or urban hospital based on its geographic location. A hospital is considered a teaching hospital if it has one or more Accreditation Council for Graduate Medical Education (ACGME)-accredited residency programs, is a member of the Council of Teaching Hospitals (COTH), or has a resident physician to patients ratio of 0.25 or higher [12].

Study population and statistical analysis

Stata IC v16.1 (StataCorp, College Station, TX) was used for the data analysis in our study. Any patients under the age of 18 were excluded from the study. TAVR population was defined using the ICD-9 (3505, 3506) and ICD-10 (02RF38H, 02RF38Z, 02RF3JH, 02RF3KH, 02RF3KZ, 02RF37Z, 02RF37H, 02RF3JZ) procedure codes. TAVR patients were divided into two groups: with and without PD (combined AP and/or PE). The ICD codes for our study are provided in Table 1. The infectious and autoimmune pericarditis were excluded from the study. The baseline characteristics were compared using Chi-square test and student t-test for categorical and continuous variables, respectively. Any variables with a p-value of 0.20 or less from the univariate logistic regression were included in the multivariate logistic regression to find independent predictors of PD in TAVR patients. To account for inflation, each year’s total hospital costs were adjusted to an equivalent value of January 2018 using the consumer price index (CPI) from the United States Bureau of Labor Statistics [14]. Our study was exempt from the Institutional Review Board (IRB) since the NIS database does not contain any patient identifying information.

**Table 1:** Supplemental table showing ICD codes ICD: International Classification of Disease.

Comorbidities or procedures	ICD-9 codes	ICD-10 codes
Acute pericarditis (infectious and autoimmune etiologies excluded)	42091, 42090, 42099	I30, I300, I301, I308, I309
Pericardial effusion	4239	I313
Coronary artery disease (CAD)	414.x	I25.x
Acute myocardial infarction (AMI)	410.x, 412.x	I21.x, I22.x, I25.2
Congestive heart failure (CHF)	398.91, 402.01, 402.11, 402.91, 404.01, 404.03, 404.11, 404.13, 404.91, 404.93, 425.4-425.9, 428.x	I09.9, I11.0, I13.0, I13.2, I25.5, I42.0, I42.5-I42.9, I43.x, I50.x, P29.0
Diabetes with and without complications	250.0-250.3, 250.4-250.9	E10.0, E10.1, E10.9, E11.0, E11.1, E11.9, E12.0, E12.1, E12.9, E13.0, E13.1, E13.9, E14.0, E14.1, E14.9, E10.2-E10.8, E11.2-E11.8, E12.2-E12.8, E13.2-E13.8, E14.2-E14.8
Hypertension (HTN)	401.x, 402.x-405.x	I10.x, I11.x-I13.x, I15.x
Hyperlipidemia (HLD)	272.x	E78.x
Atrial fibrillation (Afib)	42731	I48.0-I48.2, I48.91
Atrial flutter (Aflutter)	42732	I48.3, I48.4, I48.9, I48.92
Cerebrovascular disease (CVD)	362.34, 430.x-438.x	G45.x, G46.x, H34.0, I60.x-I69.x
Peripheral vascular disease (PVD)	093.0, 437.3, 440.x, 441.x, 443.1-443.9, 447.1, 557.1, 557.9, V43.4	I70.x, I71.x, I73.1, I73.8, I73.9, I77.1, I79.0, I79.2, K55.1, K55.8, K55.9, Z95.8, Z95.9
Chronic obstructive pulmonary disease (COPD)	416.8, 416.9, 490.x-505.x, 506.4, 508.1, 508.8	I27.8, I27.9, J40.x-J47.x, J60.x-J67.x, J68.4, J70.1, J70.3
Pulmonary hypertension		
Coagulopathy	286.x, 287.1, 287.3-287.5	D65-D68.x, D69.1, D69.3-D69.6
Cirrhosis	570.x, 571.x, 572.2-572.8, 573.3, 573.4, 573.8, 573.9	K70.x, K71.1, K71.3-K71.5, K71.7, K72.x-K74.x
Thyroid disease	240.x- 244.x	E0.1x-E0.4x
Chronic kidney disease 3/4 (CKD 3/4)	5854, 5853, 40413	N183, N184, N189
End-stage renal disease (ESRD)	403.01, 403.11, 403.91, 404.02, 404.03, 404.12, 404.13, 404.92, 404.93, 585.x, 586.x, 588.0, V42.0, V45.1, V56.x	I12.0, I13.1, N18.x, N19.x, N25.0, Z49.0-Z49.2, Z94.0, Z99.2
Malignancy	140.x-172.x, 174.x-195.8, 200.x-208.x, 238.6, 196.x-199.x	C00.x-C26.x, C30.x-C34.x, C37.x-C41.x, C43.x, C45.x-C58.x, C60.x-C76.x, C81.x-C85.x, C88.x, C90.x-C97.x, C77.x-C80.x
Malnutrition	262.x-263.x	E44.x-E46.x
Systemic lupus erythematosus (SLE)	7100, 6954	M329, M3210, M3211, M3213, M3212, M3214, M3215, M3219, M328, M321
Rheumatoid disease	446.5, 710.0-710.4, 714.0-714.2, 714.8, 725.x	M05.x, M06.x, M31.5, M32.x-M34.x, M35.1, M35.3, M36.0
Procedures		
Percutaneous coronary intervention (PCI)	0066, 3601, 3602, 3605, 3606, 3607	027234Z, 0270346, 027034Z, 02703D6, 02703DZ, 02703Z6, 02703ZZ, 0270446, 027044Z, 02704D6, 02704DZ, 02704Z6, 02704ZZ, 0271346, 027134Z, 02713D6, 02713DZ, 02713Z6, 02713ZZ, 0271446, 027144Z, 02714D6, 02714DZ, 02714Z6, 02714ZZ, 02772346, 027234Z, 02723D6, 02723DZ, 02723Z6, 02723ZZ, 0272446, 027244Z, 02724D6, 02724DZ, 02724Z6, 02724ZZ, 0273346, 027334Z, 02733D6, 02733DZ, 02733Z6, 02733ZZ, 0273446, 027344Z, 02734D6, 02734DZ, 02734Z6, 02734ZZ
Coronary artery bypass grafting (CABG)	3610610, 3611, 3612, 3613, 3614, 3615, 3616, 3617, 3619	02120AC, 0211089, 0211088, 02100JW, 02100JC, 02100JF, 02100J3, 02100J9, 02130KW, 02130KC, 02130KF, 02130K3, 02130K8, 02130K9, 02100K9, 02100K8, 02100KW, 02100KF, 02100KC, 02130J3 02130J9, 02130J8, 02130JW, 02130JC, 02130JF, 02110JW, 02110A3, 021009W, 0210098, 02120JW, 02120JC, 02120JF, 02100Z8, 02120J8, 02120J3, 02120J9, 02110ZF, 02110ZC, 02110Z9, 02110Z8, 02110Z3, 02130AF, 0213093, 0213098, 0213099, 021309C, 021309F, 021309W, 02120KW, 02120KC, 02120KF, 02100AW, 02120AF, 02100K3, 021308F, 021308C, 021308W, 0213089, 0213088, 0213083, 02120A9, 02120A8, 02120A3, 02120AW, 02110JF, 02100Z3, 02100Z9, 02100ZC, 02100ZF, 021209C, 0212099, 02120K9, 02120K8, 02120K3, 02100A3, 02100A9, 02100A8, 02100AC, 02100AF, 0211093, 0211099, 021109W,021109C, 021109F, 0210089, 02110J3, 021008F, 02130AC"02130AW, 02130A8, 02130A9, 02130Z9, 02130Z8, 02130ZC, 02130ZF, 0212083, 0212088, 0212089, 021208W, 021208C, 021208F, 021108W, 021108C, 021108F, 0211083, 02110AC, 02110AF, 02110AW, 02110A8, 02110A9, 0211098, 02130A3, 02100J8, 021209W, 021209F, 0212093, 0212098, 02110KC, 02110KW, 02110K8, 02110K9, 02110K3, 02120Z8, 02120Z9, 02120Z3, 02120ZC, 02120ZF, 02130Z3, 0210088, 0210083, 021008W, 021008C, 02110J9, 02110J8, 02110JC, 02110KF, 021009C, 021009F, 0210099, 0210093
Pacemaker (PM) implantation	3780780, 3781, 3782, 3783	0JH604Z, 0JH634Z, 0JH804Z, 0JH834Z, 0JH605Z, 0JH835Z, 0JH606Z, 0JH636Z, 0JH806Z, 0JH836Z, 0JH805Z, 0JH635Z
Postoperative complications		
Cardiac tamponade	4233233	I314
Postoperative cardiogenic shock	99801	T8111XA
Vasopressors requirement	0017	3E030XZ, 3E033XZ, 3E040XZ, 3E043XZ, 3E050XZ, 3E053XZ, 3E060XZ, 3E063XZ
Mechanical ventilation requirement	9670, 9671, 9672	5A1955Z, 5A1945Z, 5A1935Z

Outcomes

The primary outcome of interest was in-hospital mortality in TAVR patients with PD. The secondary outcomes of interest were the mean length of stay, mean total hospital cost, and postoperative complications (cardiac tamponade, postoperative cardiogenic shock, vasopressors, and mechanical ventilation requirements).

## Results

**Figure 1:**
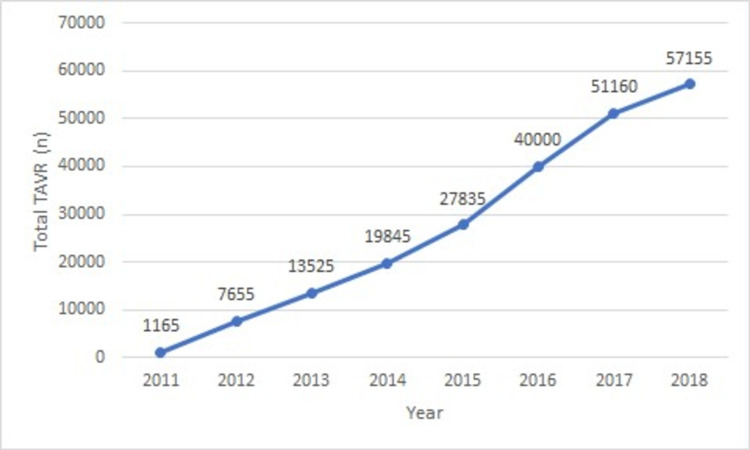
Trend of transcatheter aortic valve repair (TAVR) during 2011-2018

**Figure 2:**
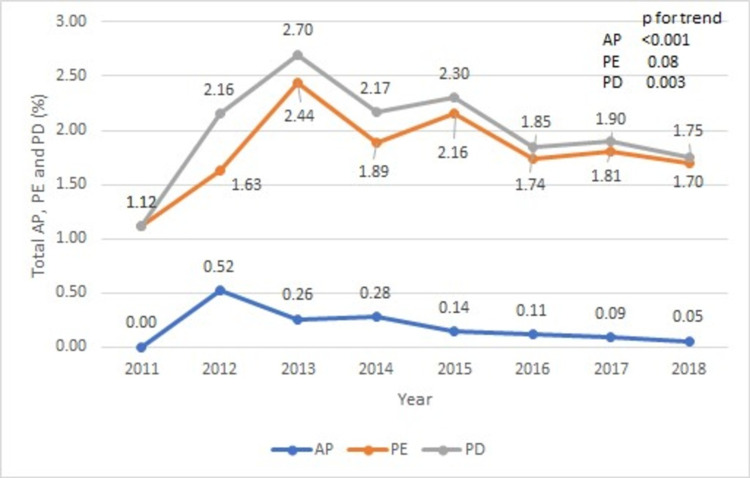
Trend of PD – AP and/or PE – during TAVR hospitalizations AP: Acute pericarditis; PE: pericardial effusion; PD: pericardial disease; TAVR: transcatheter aortic valve repair.

In our study, a total of 218,340 TAVR hospitalizations occurred over the period of eight years (2011-2018). Out of these, 4,323 (1.2%) had a concurrent diagnosis of PD (AP and/or PE). The number of TAVR hospitalizations grew exponentially from 1,165 in 2011 to 57,155 in 2018 (Table 2 and Figure 1). Similarly, the percentage of PD increased from 1.12% (n = 13) to a peak of 2.70% in 2013 and then trended down to 1.75% (n = 1000) in 2018 (Table 2 and Figure 2; p for trend = 0.003). The majority of the PD group was represented by PE (Figure 2).

**Table 2:** Number of TAVR and concurrent PD from 2011 to 2018 TAVR: Transcatheter aortic valve repair; AP: acute pericarditis; PE: pericardial effusion; PD: pericardial disease.

Year	TAVR (n)	AP (n)	PE (n)	PD (combined AP and PE, n)
2011	1165	0	13	13
2012	7655	40	125	165
2013	13525	35	330	365
2014	19845	55	375	430
2015	27835	40	600	640
2016	40000	45	695	740
2017	51160	45	925	970
2018	57155	30	970	1000
Total	218,340	290	4,033	4,323

Baseline characteristics

**Figure 3:**
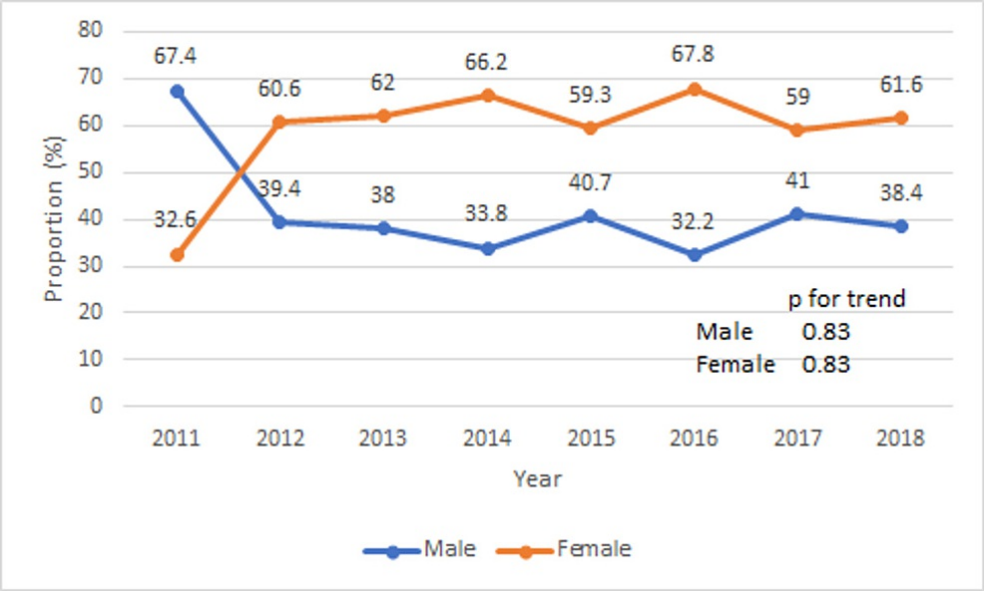
Trend of proportion of female and male (%) in PD group during TAVR hospitalizations TAVR: Transcatheter aortic valve repair; PD: pericardial disease.

**Figure 4:**
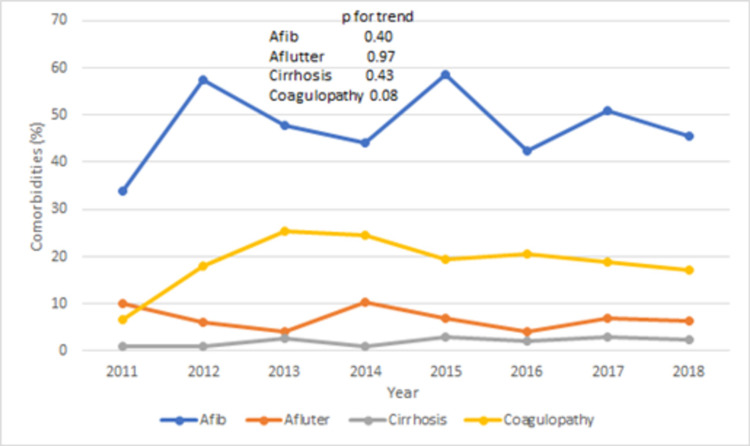
Trend of comorbidities (%) in PD group during TAVR hospitalizations TAVR: Transcatheter aortic valve repair; PD: pericardial disease.

Baseline characteristics with and without PD groups were also compared (Table 3). Compared to TAVR patients without PD, the group with PD was older (80 ± 6 vs 81±7 years, p < 0.05) and more likely to be female (46 vs 62%, p < 0.001). The PD group was more likely to be white (84.2 vs 82.9%, p = 0.83), have Medicare as a primary expected payer (90 vs 89.8%, p = 0.97), and have higher burden of comorbidities (Charlson index score ≥ 3: 55 vs 53%, p = 0.63). The proportion of females in the PD group always remained higher except in 2011 (Figure 3, p for trend = 0.83). The PD group had a higher burden of comorbidities [Afib: 48.7 vs 40.5%, p < 0.001; atrial flutter (Aflutter): 6.4 vs 4%, p = 0.001; pulmonary hypertension: 17.3 vs 13.8%, p = 0.01; coagulopathy: 20 vs 13.3%, p < 0.001; malnutrition: 7.2 vs 3.1%, p < 0.001; rheumatoid disease: 5.3 vs 4%, p = 0.05]. The trend of comorbidities in the PD group remained stable with Afib being the most common comorbidity throughout the study period (Figure 4, Afib trend increased from 34% in 2011 to 45.4% in 2018, p for trend = 0.40). Similarly, the procedure’s burden was also higher in the PD group [percutaneous coronary intervention (PCI): 4.6 vs 2.8%, p < 0.001; coronary artery bypass grafting (CABG): 0.81 vs 0.30%, p = 0.02; PM implantation: 13.5 vs 9%, p < 0.001].

**Table 3:** Baseline characteristics of with and without PD groups in TAVR population TAVR: Transcatheter aortic valve repair; PD: pericardial disease; CAD: coronary artery disease; AMI: acute myocardial infarction; CHF: congestive heart failure; HTN: hypertension; HLD: hyperlipidemia; Afib: atrial fibrillation; Aflutter: atrial flutter; CVD: cerebrovascular disease; PVD: peripheral vascular disease; COPD: chronic obstructive pulmonary disease; CKD: chronic kidney disease; ESRD: end-stage renal disease; SLE: systemic lupus erythematosus; PCI: percutaneous coronary intervention; CABG: coronary artery bypass grafting; PM: pacemaker.

	Total TAVR (n = 218,340)		
Baseline characteristics	With PD (n = 4323, 1.2%)	Without PD (n = 214,017, 98.8%)	p value
Age (years; mean ± SD)	81 ± 7	80 ± 6	<0.05
Sex			<0.001
Male	1643 (38%)	115,569 (54%)	
Female	2680 (62%)	98,448 (46%)	
Race			0.83
White	3640 (84.2%)	117,420 (82.9%)	
African American	117 (2.7%)	8560 (4%)	
Hispanic	208 (4.8%)	9417 (4.4%)	
Others	358 (8.3%)	18620 (8.7%)	
Hospital bed size			0.33
Small	303 (7%)	13269 (6.2%)	
Medium	907 (21%)	40663 (19%)	
Large	3113 (72%)	160085 (74.8%)	
Hospital location/teaching status			0.69
Rural	61 (1.4%)	1712 (0.8%)	
Urban nonteaching	424 (9.8%)	20118 (9.4%)	
Urban teaching	3838 (88.8%)	192187 (89.8%)	
Primary expected payer			0.97
Medicare	3891 (90%)	192187 (89.8%)	
Medicaid	65 (1.5%)	2568 (1.2%)	
Private insurance	282 (6.5%)	14981 (7%)	
Others	85 (2%)	4280 (2%)	
Median household income for patient's ZIP code			0.16
0-25th percentile	908 (21%)	45585 (21.3%)	
26th to 50th percentile	968 (22.4%)	54574 (25.5%)	
51st to 75th percentile	1111 (25.7%)	56715 (26.5%)	
76th to 100th percentile	1336 (30.9%)	57143 (26.7%)	
Charlson comorbidity index score			0.63
0	398 (9.2%)	21402 (10%)	
1	770 (17.8%)	36383 (17%)	
2	778 (18%)	42803 (20%)	
≥3	2378 (55%)	113429 (53%)	
Comorbidities			
CAD	2594 (60%)	147672 (69%)	<0.001
AMI	519 (12%)	32103 (15%)	0.04
CHF	3134 (72.5%)	153236 (71.6%)	0.71
Diabetes with and without complications	1236 (28.6%)	78758 (36.8%)	<0.001
HTN	1362 (31.5%)	72124 (33.7%)	0.11
HLD	2581 (59.7%)	143391 (67%)	<0.001
Afib	2105 (48.7%)	86677 (40.5%)	<0.001
Aflutter	277 (6.4%)	8561 (4%)	0.001
CVD	588 (13.6%)	24398 (11.4%)	0.11
PVD	1038 (24%)	46014 (21.5%)	0.23
COPD	1236 (28.6%)	62065 (29%)	0.83
Pulmonary hypertension	748 (17.3%)	29534 (13.8%)	0.01
Coagulopathy	866 (20%)	28464 (13.3%)	<0.001
Cirrhosis	108 (2.5%)	3424 (1.6%)	0.16
Thyroid disease	1020 (23.6%)	44302 (20.7%)	0.12
CKD3/4	1211 (28%)	56501 (26.4%)	0.13
ESRD	208 (4.8%)	8561 (4%)	0.10
Malignancy	221 (5.1%)	9203 (4.3%)	0.40
Malnutrition	311 (7.2%)	6635 (3.1%)	<0.001
SLE	10 (0.23%)	792 (0.37%)	0.78
Rheumatoid disease	229 (5.3%)	8561 (4%)	0.05
Procedures			
PCI	199 (4.6%)	5993 (2.8%)	<0.001
CABG	35 (0.81%)	642 (0.30%)	0.02
PM implantation	584 (13.5%)	19262 (9%)	<0.001

Univariate and multivariate predictors of PD in TAVR

The univariate and multivariate predictors of PD in TAVR patients are shown in Tables 4, 5. The univariate predictors are age >75, female sex, medium hospital bed size, rural hospital location, Afib, Aflutter, cerebrovascular disease (CVD), peripheral vascular disease (PVD), pulmonary hypertension, coagulopathy, cirrhosis, thyroid disease, end-stage renal disease (ESRD), malnutrition, rheumatoid disease, PCI, CABG, and PM implantation. The multivariate predictors are age >75 [adjusted odds ratio (aOR) 1.34, 95% confidence interval (CI) 1.13-1.60, p = 0.001], female sex (aOR 1.96, 95% CI 1.70-2.26, p < 0.001), Afib (aOR 1.30, 95% CI 1.14-1.50, p < 0.001), Aflutter (aOR 1.44, 95% CI 1.08-1.91, p = 0.01), PVD (aOR 1.19, 95% CI 1.09-1.40, p = 0.03), coagulopathy (aOR 1.52, 95% CI 1.28-1.81, p < 0.001), cirrhosis (aOR 1.62, 95% CI 1.04-2.53, p = 0.03), malnutrition (aOR 2.03, 95% CI 1.55-2.66, p < 0.001), PCI (aOR 1.59, 95% CI 1.15-2.21, p = 0.005), CABG (aOR 2.81, 95% CI 1.28-6.14, p = 0.01), and PM implantation (aOR 1.56, 95% CI 1.28-1.90, p < 0.001).

**Table 4:** Univariate predictors of PD in patients undergoing TAVR TAVR: Transcatheter aortic valve repair; PD: pericardial disease; CAD: coronary artery disease; AMI: acute myocardial infarction; CHF: congestive heart failure; HTN: hypertension; HLD: hyperlipidemia; Afib: atrial fibrillation; Aflutter: atrial flutter; CVD: cerebrovascular disease; PVD: peripheral vascular disease; COPD: chronic obstructive pulmonary disease; CKD: chronic kidney disease; ESRD: end-stage renal disease, SLE: systemic lupus erythematosus; PCI: percutaneous coronary intervention; CABG: coronary artery bypass grafting; PM: pacemaker.

Predictors	TAVR with PD (n = 4,323)	
	Univariate odds ratio OR (95% confidence interval, CI)	p value
Age group		
18-45	0.37 (0.052-2.69)	0.33
46-60	0.83 (0.51-1.33)	0.44
61-75	0.72 (0.60-0.86)	<0.001
>75	1.39 (1.17-1.65)	<0.001
Female vs male	1.90 (1.66-2.17)	<0.001
Race		
White	1.10 (0.91-1.33)	0.31
African American	0.65 (0.43-0.99)	0.04
Hispanic	1.07 (0.78-1.47)	0.64
Others	0.95 (0.73-1.23)	0.72
Hospital bed size		
Small	1.12 (0.83-1.52)	0.43
Medium	1.13 (0.95-1.35)	0.14
Large	0.86 (0.73-1.01)	0.07
Hospital location/teaching status		
Rural	1.71 (0.95-3.06)	0.07
Urban nonteaching	1.03 (0.80-1.33)	0.78
Urban teaching	0.91 (0.72-1.15)	0.44
Primary expected payer		
Medicare	1.01 (0.81-1.27)	0.87
Medicaid	1.24 (0.71-2.16)	0.44
Private insurance	0.93 (0.71-1.22)	0.63
Others	0.97 (0.59-1.59)	0.93
Comorbidities		
CAD	0.66 (0.57-0.76)	<0.001
AMI	0.77 (0.62-0.95)	0.01
CHF	1.04 (0.89-1.21)	0.60
Diabetes with and without complications	0.69 (0.59-0.80)	<0.001
HTN	0.89 (0.77-1.03)	0.14
HLD	0.72 (0.62-0.83)	<0.001
Afib	1.39 (1.22-1.59)	<0.001
Aflutter	1.61 (1.23-2.12)	0.001
CVD	1.21 (0.98-1.48)	0.06
PVD	1.14 (0.97-1.35)	0.09
COPD	0.98 (0.84-1.13)	0.81
Pulmonary hypertension	1.30 (1.09-1.56)	0.003
Coagulopathy	1.61 (1.37-1.91)	<0.001
Cirrhosis	1.50 (0.98-2.31)	0.06
Thyroid disease	1.18 (1-1.39)	0.04
CKD3/4	1.09 (0.93-1.26)	0.25
ESRD	1.21 (0.89-1.65)	0.20
Malignancy	1.20 (0.89-1.63)	0.22
Malnutrition	2.37 (1.82-3.08)	<0.001
SLE	0.61 (0.15-2.48)	0.49
Rheumatoid disease	1.32 (0.97-1.80)	0.07
Procedures		
PCI	1.69 (1.22-2.33)	0.001
CABG	2.71 (1.26-5.83)	0.01
PM implantation	1.60 (1.31-1.94)	<0.001

**Table 5:** Multivariate predictors of PD in patients undergoing TAVR TAVR: Transcatheter aortic valve repair; PD: pericardial disease; Afib: atrial fibrillation; Aflutter: atrial flutter; PVD: peripheral vascular disease; PCI: percutaneous coronary intervention; CABG: coronary artery bypass grafting; PM: pacemaker.

Predictors	TAVR with PD (n = 4,323)	
	Multivariate odds ratio; aOR (95% CI)	p value
Age group		
>75	1.34 (1.13-1.60)	0.001
Female vs male	1.96 (1.70-2.26)	<0.001
Comorbidities		
Afib	1.30 (1.14-1.50)	<0.001
Aflutter	1.44 (1.08-1.91)	0.01
PVD	1.19 (1.09-1.40)	0.03
Coagulopathy	1.52 (1.28-1.81)	<0.001
Cirrhosis	1.62 (1.04-2.53)	0.03
Malnutrition	2.03 (1.55-2.66)	<0.001
Procedures		
PCI	1.59 (1.15-2.21)	0.005
CABG	2.81 (1.28-6.14)	0.01
PM implantation	1.56 (1.28-1.90)	<0.001

Primary and secondary outcomes

**Figure 5:**
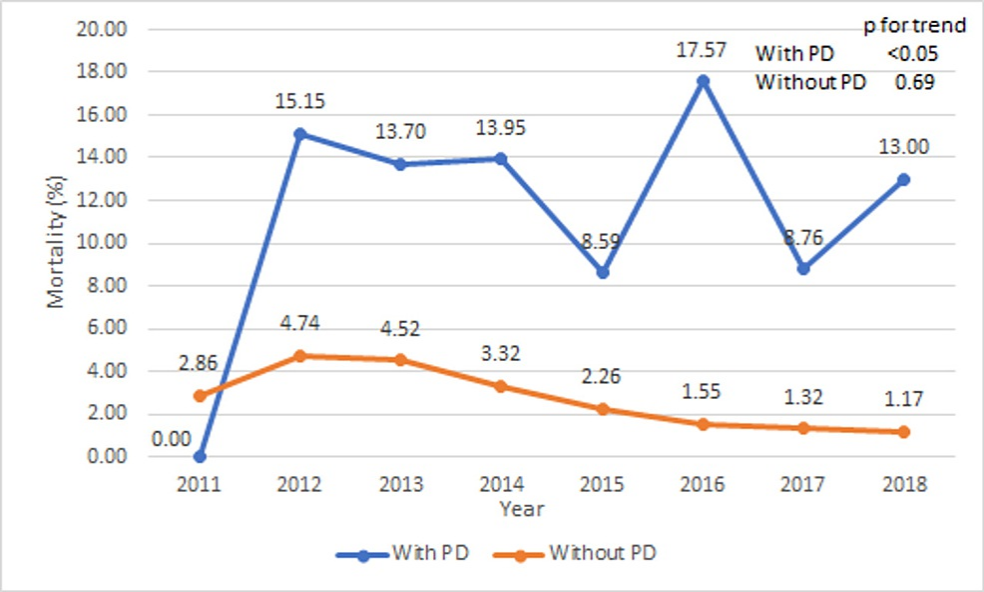
Trend of mortality (%) in with or without PD groups during TAVR hospitalizations PD: Pericardial disease; TAVR: transcatheter aortic valve repair.

**Figure 6:**
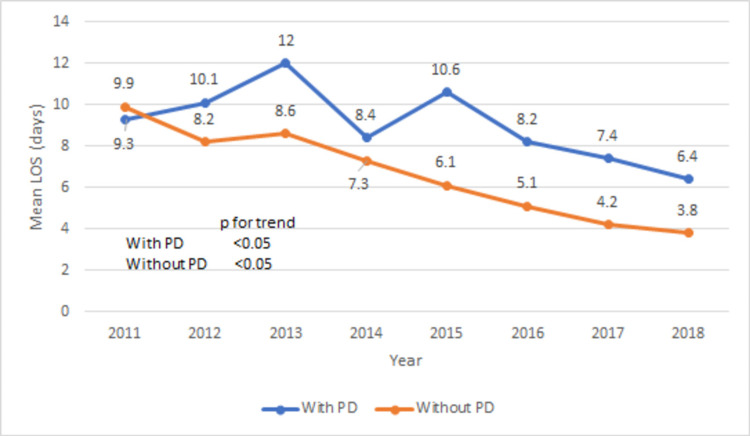
Trend of mean length of stay (in days) in with or without PD groups during TAVR hospitalizations PD: Pericardial disease; TAVR: transcatheter aortic valve repair.

**Figure 7:**
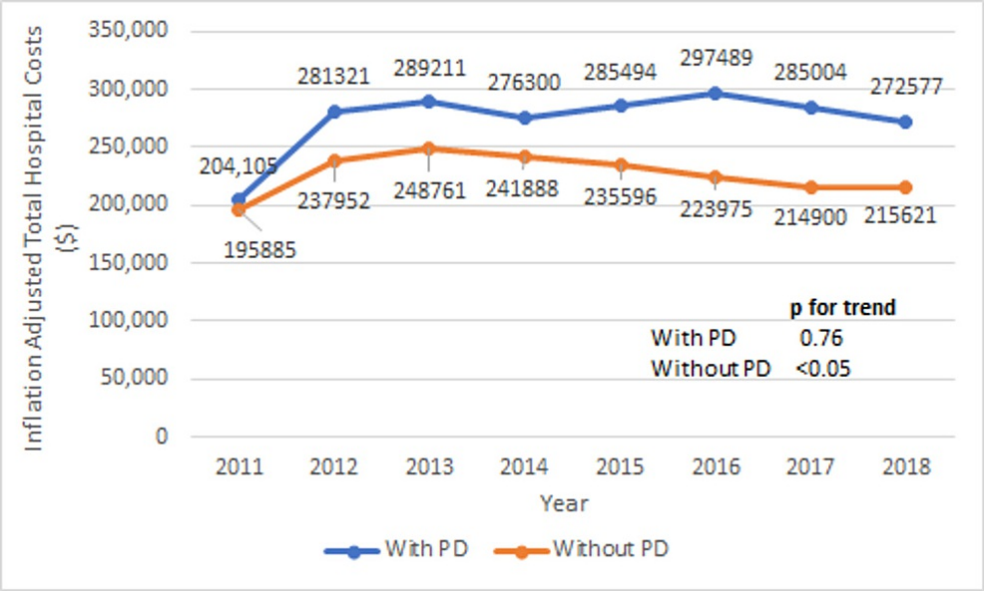
Trend of mean total hospital cost (inflation adjusted, in dollars) in with or without PD groups during TAVR hospitalizations PD: Pericardial disease; TAVR: transcatheter aortic valve repair.

The comparison of in-hospital outcomes and postoperative complications between the groups with and without PD are shown in Table 6. TAVR patients with PD have significantly higher in-hospital mortality rate (12.3 vs 1.9%, p < 0.001). The mortality rate in the PD group increased from 0% in 2011 to 17.57% in 2016 and then trended down to 13% in 2018 (Figure 5, p for trend < 0.05). The mean length of stay is also higher in the PD group (8.4 vs 5.3 days, p < 0.001). The trend of mean length of stay trended down from 9.3 days in 2011 to 6.4 days in 2018 (Figure 6, p for trend < 0.05). Similarly, mean total hospital cost is higher in the PD group ($283,389 vs $224,544, p < 0.001). The trend of mean total hospital cost is shown in Figure 7. Compared to the TAVR group without PD, TAVR group with PD has higher rates of postoperative complications (cardiac tamponade: 0.41 vs 21.5, p < 0.001), postoperative cardiogenic shock (0.45 vs 2.3%, p < 0.001), vasopressor requirements (2.1 vs 5.4%, p < 0.001), and mechanical ventilation requirement (4 vs 11.5%, p < 0.001).

**Table 6:** In-hospital outcomes and postoperative complications in TAVR patients with PD PD: Pericardial disease; TAVR: transcatheter aortic valve repair.

In-hospital outcome and postoperative complications	With PD (n = 4,323, 1.2%)	Without PD (n = 214,017, 98.8%)	p value
In-hospital mortality, n (%)	535 (12.3%)	4168 (1.9%)	<0.001
Mean length of stay (in days)	8.4	5.3	<0.001
Mean inflation-adjusted hospital cost (in US dollars)	283,389	224,544	<0.001
Cardiac tamponade, n (%)	930 (21.5%)	878 (0.41%)	<0.001
Postoperative cardiogenic shock, n (%)	100 (2.3%)	963 (0.45%)	<0.001
Vasopressor requirements, n (%)	234 (5.4%)	4494 (2.1%)	<0.001
Mechanical ventilation requirement, n (%)	497 (11.5%)	8561 (4%)	<0.001

## Discussion

Over a decade ago, AS was the most common surgically treated valvular heart disease, and SAVR historically has been the treatment of choice for patients requiring aortic valve replacement. TAVR has recently been shown to be a safe and efficacious alternative to SAVR for severe symptomatic AS in an ever-increasing number of patients. Since 2012, there has been a more than four-fold increase in TAVR procedures, and the proportion of TAVR in patients undergoing aortic valve replacement has increased from 11.9% in 2012 to 43.2% in 2016 [15,16]. This was seen again in our study where we saw a 4,291% increase in TAVR hospitalizations over the eight-year period of 2011-2018. The indications for TAVR have expanded from inoperable and high surgical risk to moderate to low surgical risk for patients now. This expansion is likely responsible for our study’s trend of the increasing number of TAVR hospitalizations and reflects an overall trend to less invasive procedures as well as more acceptance of TAVR in the medical community.

While TAVR is proving to be non-inferior and in some cases a safer option compared to SAVR for patients with severe AS, various perioperative and postoperative complications are associated with it and should not be ignored. PD is emerging as a rare but important complication of TAVR. The true incidence of AP in patients undergoing TAVR is not known at this time as the medical literature hinges solely on case reports [10,11]. However, PE as a complication from TAVR has been studied more extensively. In a study by Lange et al., out of 412 patients who underwent TAVR between 2007 and 2010, 12.8% developed pericardial effusion [17]. Interestingly, Ogunbayo et al. studied 34,820 TAVR patients using the NIS database and reported only a 1.3% prevalence of significant pericardial complications [18]. Our study statistics were similar to the latter, with our study finding a 1.2% prevalence of PD in patients undergoing TAVR, which is likely closer to the true prevalence - an important number for clinicians to know when discussing procedural risk with patients. The majority of patients with significant pericardial effusion (PE) require open surgical repair that possesses a unique challenge as many of these patients currently undergoing TAVR are already at high or inoperable surgical risk although this is a dynamic situation, given the ever-increasing indications for TAVR [19]. As for the etiology of PD, PCIS, annular, and cardiac or great vessel rupture causing ongoing inflammation and inflammatory response are the possible mechanisms for the development of pericarditis and PE after TAVR although more research is needed [9].

In a retrospective study by Ogunbayo et al., patients with pericardial complications were older (mean age 82.9) and more often female (73.1%). Female sex (OR 2.29, 95% CI 1.46-3.6, p < 0.001) and history of coagulopathy (OR 1.6, 95% CI 1.05-2.46, p = 0.031) were associated with higher odds of having pericardial complications, and the burden of certain comorbidities was also higher in this group (coagulopathy: 34.4 vs 23.5% and malnutrition: 7.5 vs 2.6%) [18]. In addition, the mortality rate and cardiogenic shock were reported to be 24.7% and 8.6%, respectively. Similarly, the PD group in our study has a higher burden of coagulopathy and malnutrition, and both female and coagulopathy were independent predictors of PD (Tables 3, 5). While both the mortality rate and cardiogenic shock of the PD group were higher (12.3 and 2.3%, respectively) in our study, they both were much lower compared to the above findings by Ogunbayo et al. [18].

Cardiac tamponade is one of the most feared and potentially catastrophic sequelae of PD. TAVR has been associated with the development of cardiac tamponade as evidenced by Rezq et al. who studied 389 patients who underwent TAVR from 2007 to 2012 and found a 4.3% incidence of cardiac tamponade [20]. This was shown again in another study of 1,957 TAVR patients, which showed the incidence of cardiac tamponade to be 2.6% [21]. Our study showed an even higher rate (21.5%) of cardiac tamponade (Table 6). This substantially higher number though was likely due to the patient population studied as all our patients already had PD placing them at higher baseline risk. This is notable in that approximately one in five patients with PE post-TAVR will develop cardiac tamponade illustrating that PE should not be trivialized by the treating cardiovascular team.

Notable risk factors were examined in our study, and we found age >75, female sex, Afib, Aflutter, PVD, coagulopathy, cirrhosis, malnutrition, and PCI as independent predictors of PD in TAVR patients (Table 5). Previous studies have reported that female and elderly patients have thinner myocardial walls that increase their risk of pericardial complications, likely explaining our finding of age and female sex being risk factors for PD [18]. Malnutrition has been previously reported as a cause of PE although most of the studies have been limited to case reports and prospective cohort studies in children [22,23]. Our study found malnutrition as an independent predictor of PD, which is notable in that it could have clinical applications in choosing suitable candidates for TAVR as well as pre-operative risk stratification (Table 5).

High-grade atrioventricular (AV) block is a complication of TAVR with wide-ranging implications. Previous studies have shown the incidence of high-grade AV block to be 2%-7% after TAVR with 85%-90% of those patients requiring PM implantation, which has additional procedural risks and complications, including PD [24,25]. Ogunbayo et al., interestingly though, reported that cardiovascular implantable electronic device (CIED) was associated with lower risk (OR 0.32, 95% CI 0.11-0.88, p = 0.028) of pericardial complications in the TAVR cohort [18]. In contrast, our study found PM implantation associated with increased odds (OR 1.56, CI 1.28-1.90, p < 0.001, 13.5 vs 9%) of PD (Table 5). Moreover, a history of CABG or previous cardiac surgery has been associated with a lower risk of pericardial complications due to pericardial inflammation and fibrosis after pericardiectomy [18,24]. Again, our study had different findings showing that CABG during TAVR hospitalization increases the odds of PD (Table 5). These findings help further elucidate the baseline characteristics of patients who are better candidates and can help clinicians better select patients in the future for the appropriate surgical intervention.

TAVR has shown to have lower all-cause mortality rates compared to SAVR in various studies and clinical trials although the mortality rate could be influenced by the complications of the TAVR procedure itself. In a study of 1,360 TAVR patients by Akinseye et al., 65 (5%) patients died during the index hospitalization [26]. In another study by Pilgrim et al., the in-hospital mortality rate for TAVR hospitalization was 2.9% [27]. The mortality rate for TAVR patients with PD has not been well studied. The mortality rate in our study cohort ranged from 0% to 17.57% during 2011-2018. The possible explanation for the 0% mortality rate in 2011 could be due to only a small number of patients developing PD in a cohort of 1,165 TAVR patients. Our study showed a very high in-hospital mortality rate (highest 17.57% in 2016, Figure 5) in TAVR patients with PD implying the importance of better patient selection and early intervention to improve overall morbidity and mortality in this group.

An important but often overlooked attribute to any surgical procedure is healthcare resource utilization. This is especially the case in developing countries that may not have access to the full gamut of healthcare resources of more developed countries. In a study by Arora et al., the mean length of stay for TAVR procedures was substantially lower compared to SAVR, and it decreased further from 6.3 days in 2012 to 4.3 days in 2015 (p < 0.0001) [28]. Minutello et al. reported the mean hospital cost of a TAVR at $181,912 in 2012 [29]. Length of stay and hospital cost for TAVR patients with PD have not been reported before. Our study showed an overall higher mean length of stay and total hospital cost in TAVR patients with PD, as opposed to those without PD (Table 6, Figures 6, 7). This signifies that a significant burden PD imposes on both the healthcare system and patients undergoing TAVR.

Future perspective

Large prospective studies are needed to further investigate the causality between TAVR and PD. Further studies with long-term follow-up are needed to assess the long-term and out-of-hospital impact of PD after the TAVR procedure.

Limitations

Our study has some limitations. First, we used ICD codes, and its accuracy relies heavily on administrative data entry into electronic health records. Second, some of the results of the study may have been affected by the expertise of the interventionist and the choice of techniques to perform TAVR, which cannot be taken into account. Third, the severity of comorbidities at the patient level may differ, which cannot be taken into account for the multivariate model. Fourth, the NIS database does not take death outside of the hospital into account, which can affect the primary outcome. Regardless of these limitations, our study draws attention to the least studied and rare complication of the TAVR procedure and calls for the need for further research in this area.

## Conclusions

PD is an important complication after TAVR. Older, white females with a higher burden of comorbidities and cardiovascular procedures are at high risk of having PD after TAVR. TAVR patients with PD have higher in-hospital mortality, mean length of stay, and total hospital cost. Sex-based disparities in the prevalence of PD after TAVR are an area of further research. As the indications for TAVR expand, better screening and careful patient selection are very important to decrease the burden of these important complications, and this requires collaboration between various specialties.
